# Supplementary material for: Optogenetic manipulation of nuclear Dorsal reveals temporal requirements and consequences for transcription
Source: Development. 2025 Mar 31;152(6):dev204706. doi: 10.1242/dev.204706 (PMC11993255; doi:10.1242/dev.204706)
Supplement: Supplementary information [file develop-152-204706-s1.pdf]

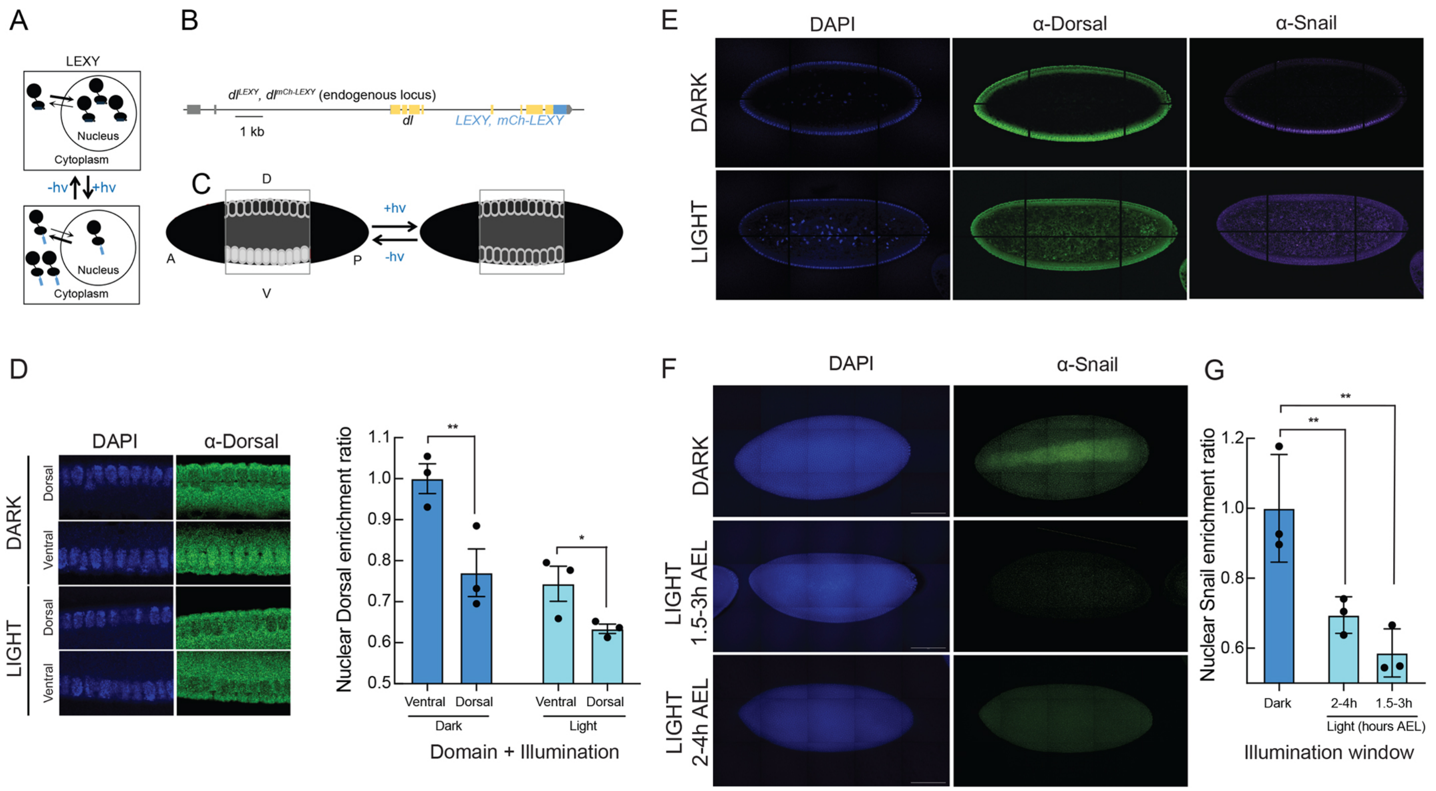

Fig. S1. Use of LEXY system for optogenetic regulation of DL nuclear localization. Related to Figures 1 and 3. **(A)** Schema of the LEXY system [19] in the dark (top) and during blue light exposure (bottom). **(B)** Diagram of the *dl-LEXY* and *dl-mCh-LEXY* loci showing insertion of the LEXY and mCh-LEXY tags (blue) into the DL (yellow) C-terminus [18]. **(C)** Schema of dark (left) and light-exposed (right) embryos demonstrating a reduction in ventral nuclei of DL^{LEXY} in the presence of light ("+hv") versus its absence ("-hv"). **(D)** Representative sagittal images of dorsal and ventral nuclei in control and light-exposed embryos showing DL nuclear levels (green) in nc14 after 2h of light exposure. Nuclear DL signal was quantified relative to ventral dark control. Under blue light, likely some low DL levels remain in the nucleus. **(E)**

Development • Supplementary information

Representative sagittal Z-planes in dark control and light-exposed embryos immunostained with anti-DL and anti-Sna antibodies demonstrating nuclear DL (green) and Sna (purple) signals are reduced upon illumination. **(F)** Representative maximum intensity projections in dark control and light-exposed embryos immunostained with anti-Sna antibody (green) and DAPI (blue) demonstrating loss of Sna protein in mid-nc14 upon illumination. Scale bar is 100um. **(G)** Quantification of Snail nuclear protein enrichment relative to dark control.

Development • Supplementary information

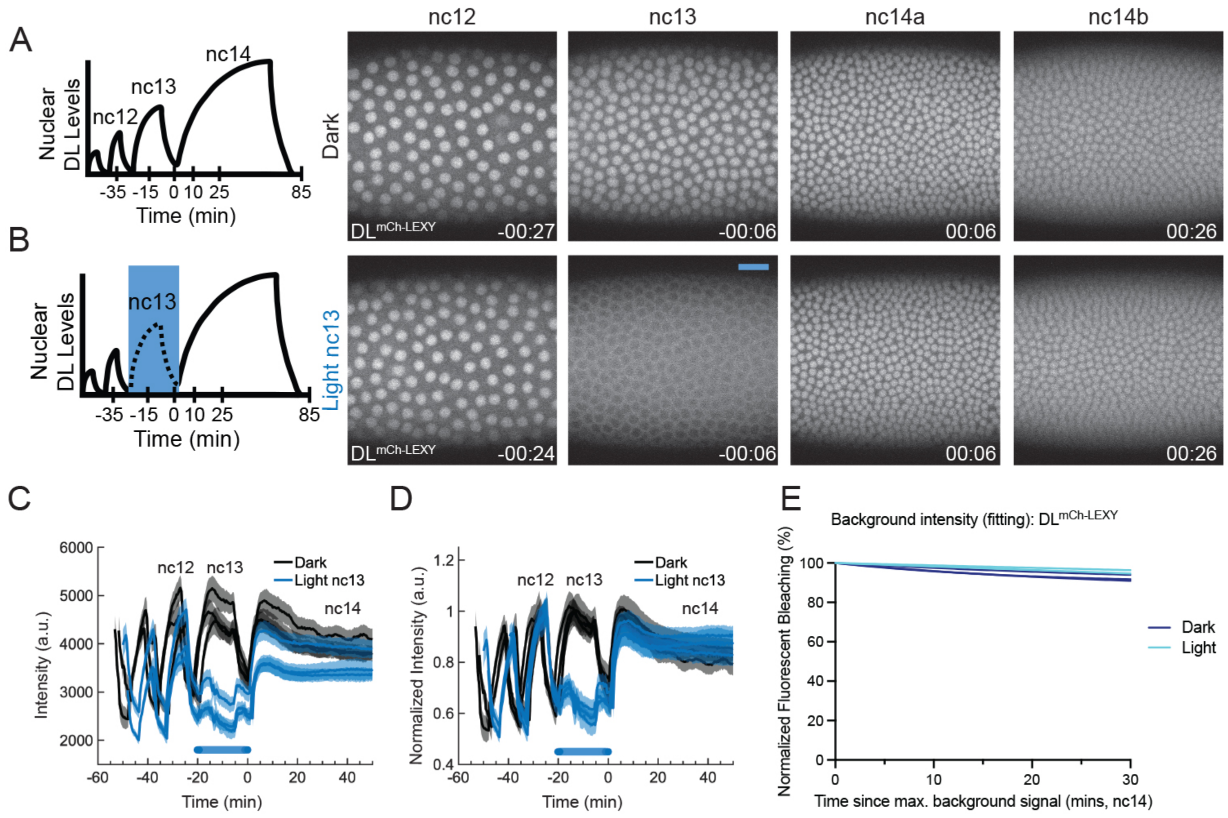

Fig. S2. Dorsal levels recover in nc14 when removed at nc13 with blue light.

Related to Figure 1. **(A-B)** DL$^{mCh\text{-}LEXY}$ signal in the dark (A) or with blue light at nc13 (B) in embryos laid by mothers homozygous for *dl-mCh-LEXY*. **(C)** Nuclei were segmented using the DL$^{mCh\text{-}LEXY}$. When no signal was present during division, the mask of the previous time point was used until DL$^{mCh\text{-}LEXY}$ signal was detected. The average intensity for nuclei were then averaged for each individual embryo and plotted. **(D)** The same as in (C) except levels were normalized by dividing by the maximum average intensity at nc12. nc12 was chosen so that embryos were normalized to the DL$^{mCh\text{-}LEXY}$ signal intensity before blue light exposure. The black lines are in the dark and the blue lines are with blue light at nc13 (mean ± s.d.). Comparing the normalized levels in nc14 between dark and light at nc13, there is little difference, suggesting that DL levels recover at nc14 when blue light is used to remove Dorsal from the nucleus at nc13. **(E)** Quantification of DL$^{mCh\text{-}LEXY}$ bleaching in living nc14 embryos for dark (black) and light-exposed (blue) embryos. N=3 embryos for all conditions.

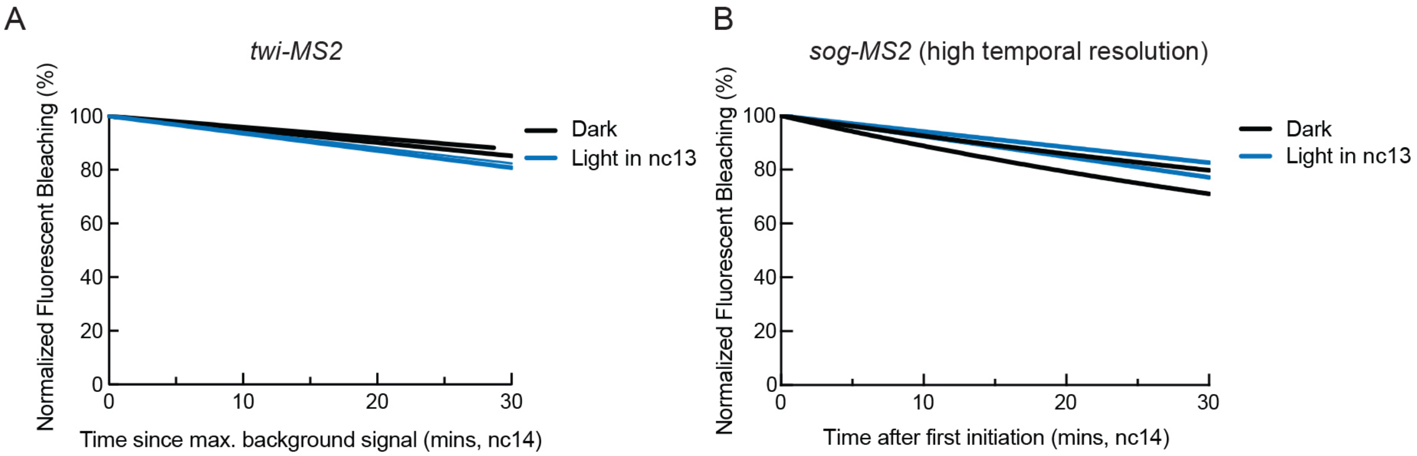

Fig. S3. Bleaching quantification during nc14 does not strongly impact signal detection. Related to figures 1 and 3. **(A)** Quantification of MCP-mCherry-NLS bleaching in living nc14 embryos for dark (black) and light-exposed (blue) embryos. **(B)** Quantification of MCP-RFPT bleaching in living nc14 embryos for dark (black) and light-exposed (blue) embryos. N=2 embryos for all conditions.

Development • Supplementary information

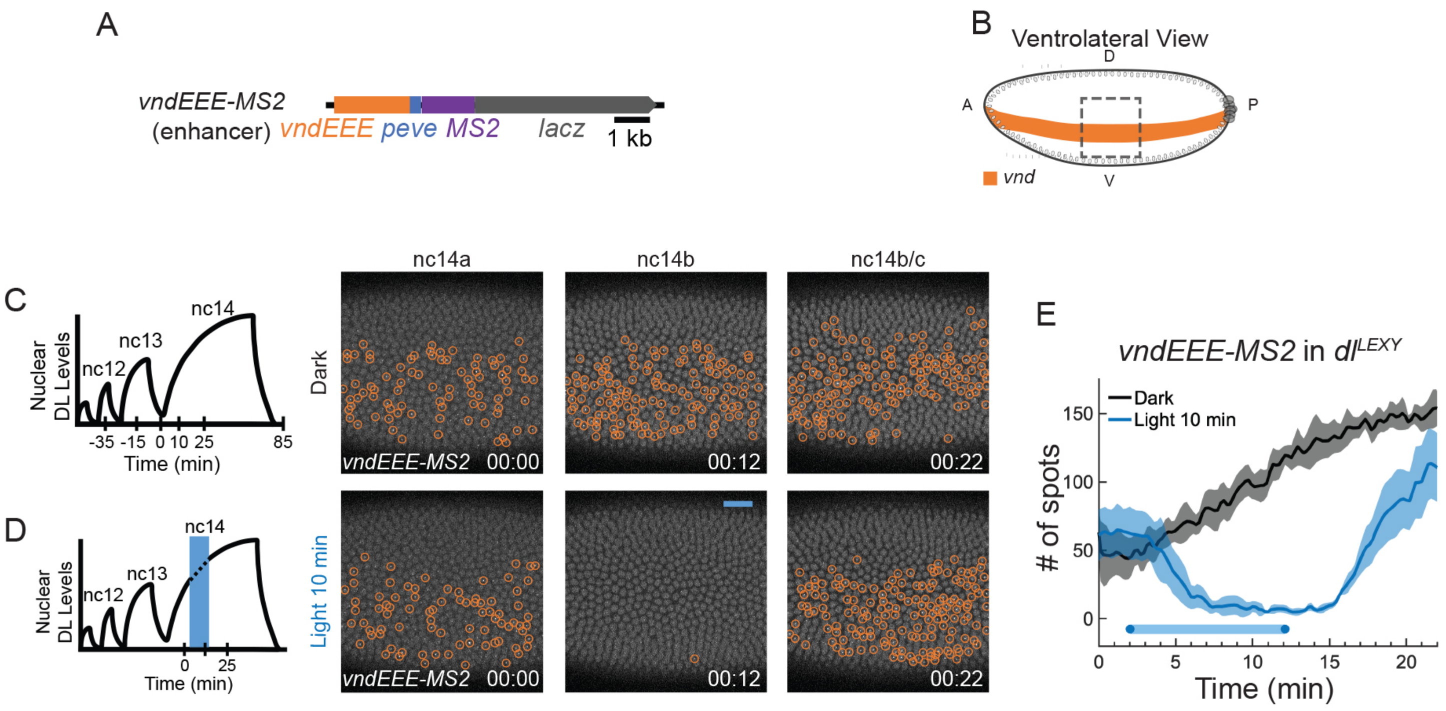

Fig. S4. *vnd* levels recover in nc14 when illuminated at nc14 for 10 min.

Related to figure 2. **(A)** The *vndEEE-MS2* reporter construct used for tracking *vnd* expression. **(B)** Schematic of the field of view used to image ventrolaterally. **(C,D)** *vndEEE-MS2* at nc14 in the dark (C) or with 10 min of blue light (D). **(E)** Quantification of the number of active TS detected for *vndEEE-MS2*. The black line is in the dark and the blue line is with blue light at nc14 (mean ± SEM; n=3 embryos for each). Foci are circled in orange for *vnd*. t=0 indicates the start of imaging in mid nc14.

Development • Supplementary information

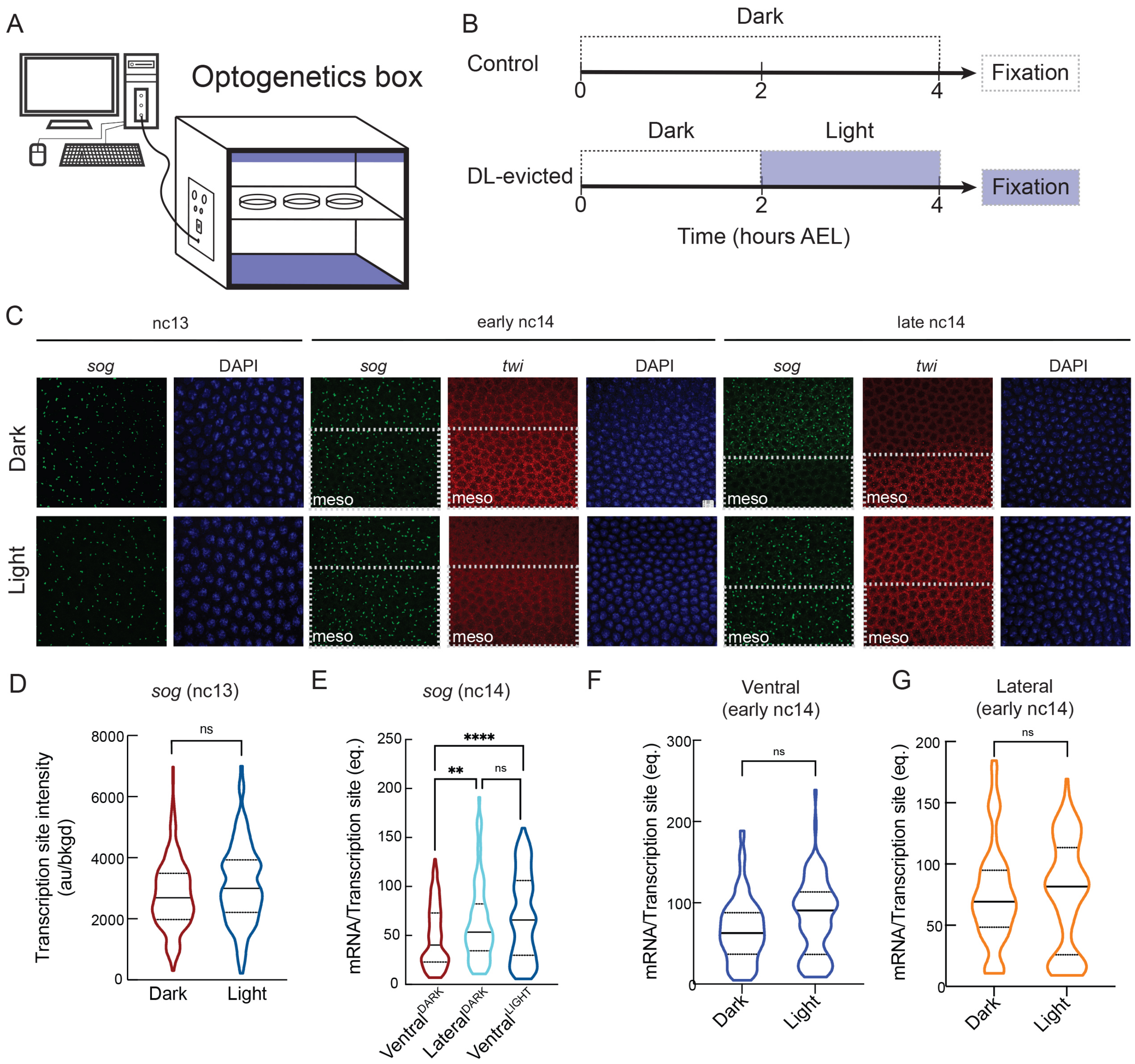

Fig. S5. Quantification of the effects of DL nuclear export on dorsoventral patterning in fixed embryos. Related to Figures 2 and 3. **(A)** Schema of custom-built optogenetic manipulation system[see 20]. **(B)** Illumination schema for control and DL-exported embryos. **(C)** Representative smiFISH maximum intensity projections of the nuclear volume for

Development • Supplementary information

nc13, nc14a and nc14c *dl-LEXY/dl-LEXY* embryos in control (dark) and DL-exported (light) conditions. Mesoderm is indicated by a dashed box. **(D)** Quantification of *sog* TS intensity for nc13 embryos in control and DL-exported conditions. **(E)** Quantification of *sog* TS intensity for the indicated domains and light conditions from a representative randomly selected sample of 200 nuclei for each domain/condition. **(F-G)** Quantification of *sog* TS intensity in early nc14 for ventral (F) and ventrolateral (G) domains. Significance determined by Kruskal-Wallis test.

Development • Supplementary information

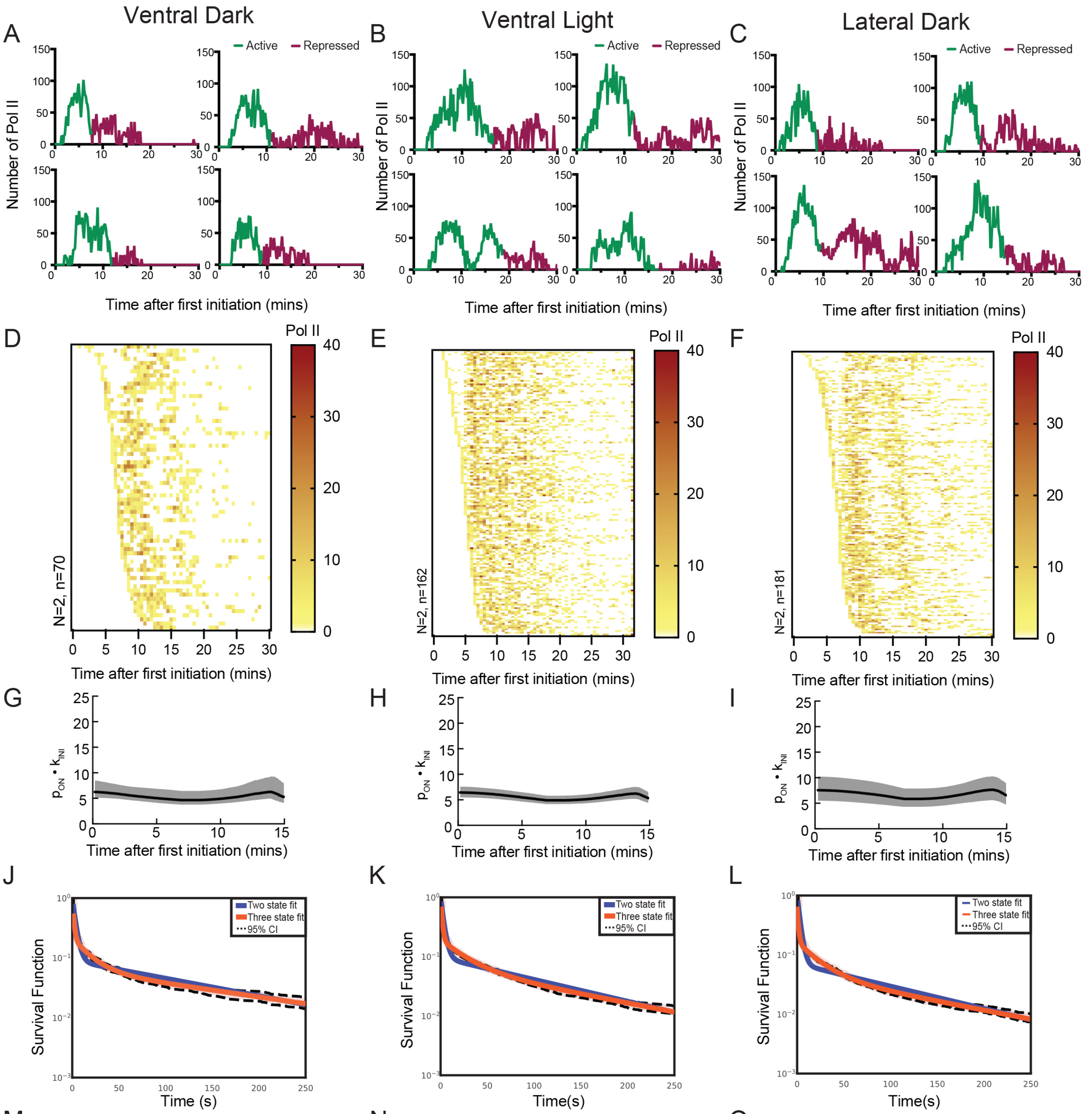

M

	ON	OFF1	OFF2
Duration (s)	7.76	179.14	15.19
Min.	7.76	179.14	15.19
Max.	7.76	179.14	15.19
Probability	0.12	0.72	0.16
Min.	0.12	0.72	0.16
Max.	0.12	0.72	0.16

N

	ON	OFF1	OFF2
Duration (s)	8.60	134.22	21.87
Min.	8.60	134.22	21.87
Max.	8.60	134.22	21.87
Probability	0.13	0.63	0.23
Min.	0.13	0.63	0.23
Max.	0.13	0.63	0.23

O

	ON	OFF1	OFF2
Duration (s)	8.86	136.38	19.23
Min.	8.86	136.38	19.23
Max.	8.86	136.38	19.23
Probability	0.15	0.60	0.25
Min.	0.15	0.60	0.25
Max.	0.15	0.60	0.25

Development • Supplementary information

Fig. S6. *sog* bursting kinetics in nc14 after DL export in nc13. Related to Figure 4. **(A-C)** Sample single nuclei *sog* transcriptional traces during the first 30 minutes of nc14 for Ventral^{DARK} (A), Ventral^{LIGHT} (B) and Lateral^{DARK} (C) regions. Trace colors indicate active (green) and repressed (purple) phases as determined by Bayesian Change Point Detection. **(D-F)** Heatmaps showing the number of polymerase initiation events for *sog* in nc14 for Ventral^{DARK} (D), Ventral^{LIGHT} (E) and Lateral^{DARK} (F) regions as a function of time. Each row represents one nucleus, and the number of Pol II initiation events per 30 s bin is indicated by bin color. **(G-I)** Kinetic parameter stability as a function of time for *sog-MS2* transcription, expressed as the product of the probability to be active (p_{ON}) and the RNA polymerase II initiation rate (k_{INI}) in nuclear cycle 14 in nc14 for Ventral^{DARK} (G), Ventral^{LIGHT} (H) and Lateral^{DARK} (I) regions. **(J-L)** Survival function for the distribution of waiting times between polymerase initiation events for *sog* transcription in nc14 for Ventral^{DARK} (J), Ventral^{LIGHT} (K) and Lateral^{DARK} (L) regions. Two-exponential fitting (blue) estimated using the Kaplan–Meyer method extends beyond the 95% confidence interval (dashed lines) and is rejected. **(M-O)** Survival function fitting indicating most parsimonious kinetic parameters and boundaries of the 95% confidence interval for the indicated domains.

Development • Supplementary information

Table S1. Reagents and Software related to Pimmett*, McGehee* et al.

REAGENT or RESOURCE	SOURCE	IDENTIFIER
**Antibodies**		
Mouse Anti-Dorsal	Developmental Studies Hybridoma Bank (DSHB)	Cat# anti-dorsal 7A4; RRID: AB_528204
Rabbit anti-Snail	This study	N/A
Alexa Fluor 488 donkey anti-mouse	Thermo Fisher Scientific	Cat# A21202; RRID: AB_141607
Alexa Fluor 647 donkey anti-rabbit	Thermo Fisher Scientific	Cat# A31573; RRID: AB_2536183
**Experimental Models: Organisms/Strains**		
D. melanogaster : *w; dl-LEXY/CyO; PrDr/TM3*	McGehee & Stathopoulos	N/A
D. melanogaster : w; *dl-mCherry-LEXY/CyO*	McGehee & Stathopoulos	N/A
D. melanogaster : *w; dl-LEXY/CyO; MCP-mCherry (w+, NLS)/TM3*	McGehee & Stathopoulos	N/A
D. melanogaster : *w; dl-LEXY; MCP-RFP* $_t$	This study	N/A
D. melanogaster : *sna-MS2 BAC (III)*	Irizarry et al	N/A
D. melanogaster : *sog-MS2 (I); Sp/CyO*	McGehee & Stathopoulos	N/A
D. melanogaster : *sog-MS2 (I)*	Whitney et al	N/A
D. melanogaster : *w; twi-MS2 (II)*	This study	N/A
D. melanogaster: w; P{w[+mC]=vndEEE-peve-24xMS2-lacZ-SV40}attP40	Falo-Sanjuan & Bray	N/A
D. melanogaster: y[1] M{w[+mC]=nanos-Cas9.P}ZH-2A w[*]		RRID:BDSC_54591
**Oligonucleotides**		
sog smiFISH probes	This study	N/A
FlapY_AF488	Integrated DNA Technologies	N/A
twi smFISH probes	LGC Biosearch Technologies	N/A
**Recombinant DNA**		
pHD-DsRed	Gratz et al	Addgene 51434
Plasmid: twi-*MS2* , DsRed homologous repair	This study	N/A
**Software and Algorithms**		
Zen 3.0 (Blue edition)	Zeiss	N/A
Zen 3.2 (Black edition)	Zeiss	N/A
Fiji/ImageJ	Schindelin et al	https://imagej.nih.gov/ij/
Prism 10	Graphpad	https://www.graphpad.com/
McGehee_2024	McGehee & Stathopoulos	https://github.com/StathopoulosLab/McGehee_2024
run_analyze_xs	Trisnadi et al.	https://www.sciencedirect.com/science/article/pii/S1046202312002629?via%3Dihub
OptoTRACK	This study	https://github.com/ant-trullo/OptoTrack
BCPD analysis	Pimmett et al; Adams & MacKay	https://github.com/mariadouaihy/BCPD_inhomogeneous_transcriptional_signal
BurstDECONV	Douaihy et al	https://github.com/oradules/BurstDECONV
Oligostan	Tsanov et al	https://bitbucket.org/muellerflorian/fish_quant/src/master/
smFisher	Dufourt et al	https://github.com/ant-trullo/smFiSH_software<br>https://github.com/ant-trullo/StripesAnalysisStudy

Development • Supplementary information

Supplemental References

Adams, R. P. and MacKay, D. J. C. (2007). Bayesian Online Changepoint Detection.

Douaihy, M., Topno, R., Lagha, M., Bertrand, E. and Radulescu, O. (2023). BurstDECONV: a signal deconvolution method to uncover mechanisms of transcriptional bursting in live cells. *Nucleic Acids Res.* 51, e88.

Dufourt, J., Bellec, M., Trullo, A., Dejean, M., De Rossi, S., Favard, C. and Lagha, M. (2021). Imaging translation dynamics in live embryos reveals spatial heterogeneities. *Science* 372, 840–844.

Falo-Sanjuan, J. and Bray, S. (2022). Notch-dependent and -independent transcription are modulated by tissue movements at gastrulation. *eLife* 11, e73656.

Gratz, S. J., Ukken, F. P., Rubinstein, C. D., Thiede, G., Donohue, L. K., Cummings, A. M. and O'Connor-Giles, K. M. (2014). Highly Specific and Efficient CRISPR/Cas9-Catalyzed Homology-Directed Repair in *Drosophila*. *Genetics* 196, 961–971.

Irizarry, J., McGehee, J., Kim, G., Stein, D. and Stathopoulos, A. (2020). Twist-dependent ratchet functioning downstream from Dorsal revealed using a light-inducible degron. *Genes Dev.* 34, 965–972.

McGehee, J. and Stathopoulos, A. (2024). Target gene responses differ when transcription factor levels are acutely decreased by nuclear export versus degradation. *Development* 151, dev202775.

Pimmett, V. L., Douaihy, M., Maillard, L., Trullo, A., Radulescu, O. and Lagha, M. (2024). Dissecting the dynamics of coordinated active transcriptional repression in a multicellular organism.

Schindelin, J., Arganda-Carreras, I., Frise, E., Kaynig, V., Longair, M., Pietzsch, T., Preibisch, S., Rueden, C., Saalfeld, S., Schmid, B., et al. (2012). Fiji: an open-source platform for biological-image analysis. *Nat. Methods* 9, 676–682.

Trisnadi, N., Altinok, A., Stathopoulos, A. and Reeves, G. T. (2013). Image analysis and empirical modeling of gene and protein expression. *Methods San Diego Calif* 62, 68–78.

Tsanov, N., Samacoits, A., Chouaib, R., Traboulsi, A.-M., Gostan, T., Weber, C., Zimmer, C., Zibara, K., Walter, T., Peter, M., et al. (2016). smiFISH and FISH-quant – a flexible single RNA detection approach with super-resolution capability. *Nucleic Acids Res.* 44, e165.

Whitney, P. H., Shrestha, B., Xiong, J., Zhang, T. and Rushlow, C. A. (2022). Shadow enhancers modulate distinct transcriptional parameters that differentially effect downstream patterning events. *Development* 149, dev200940.

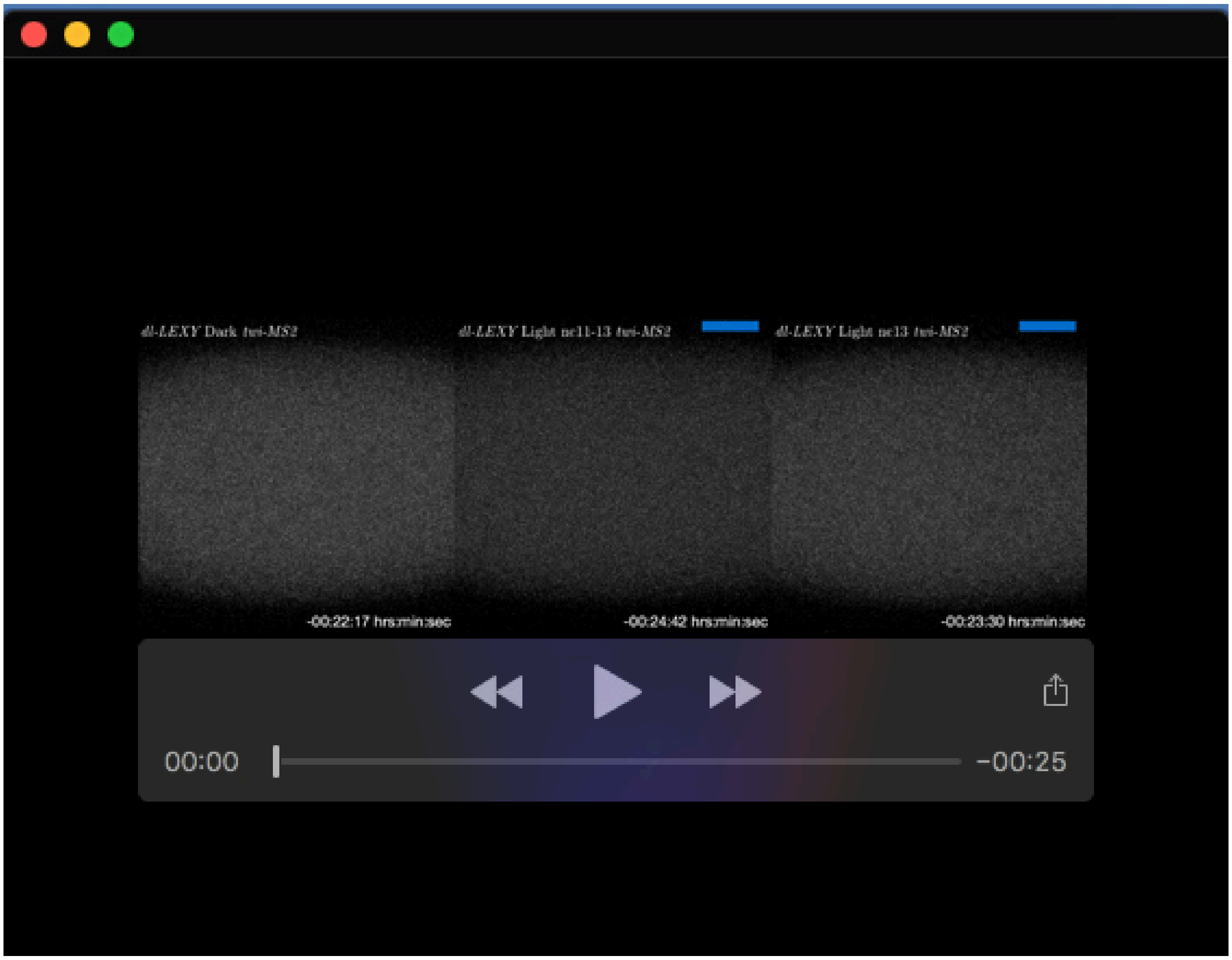

Movie 1. *twi-MS2* (magenta) in *dl-LEXY* in the dark, with light at nc11-13, and with light at nc13. Related to Fig. 1. A ventral view is shown in all panels. Nascent transcription was only detected above a threshold for Movies 1-3, and spots can only reliably be discerned before the embryo initiates gastrulation.

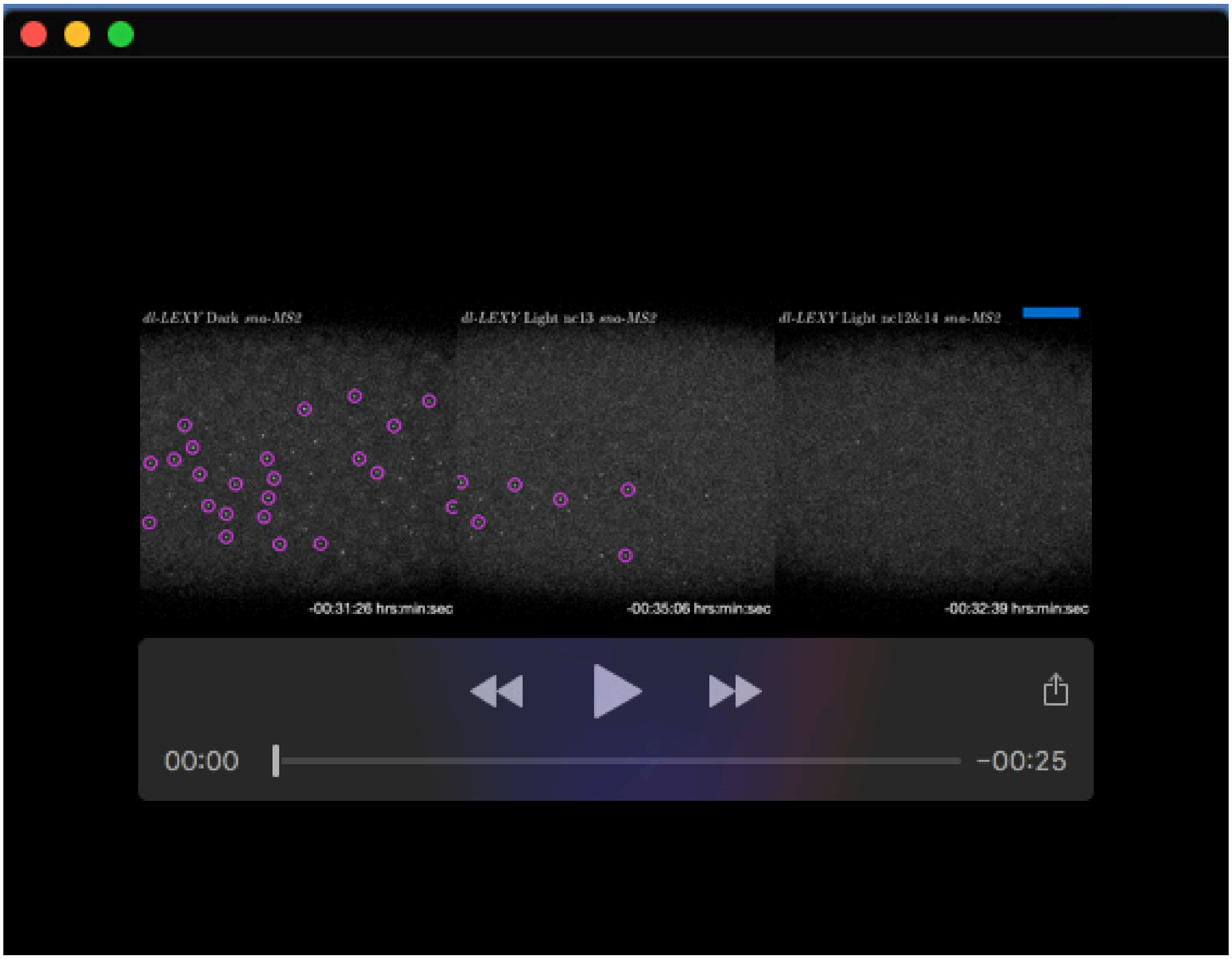

Movie 2. *sna-MS2* (magenta) in *dl-LEXY* in the dark, with light at nc13, and with light at nc12 & 14. Related to Fig. 1. A ventral view is shown in all panels.

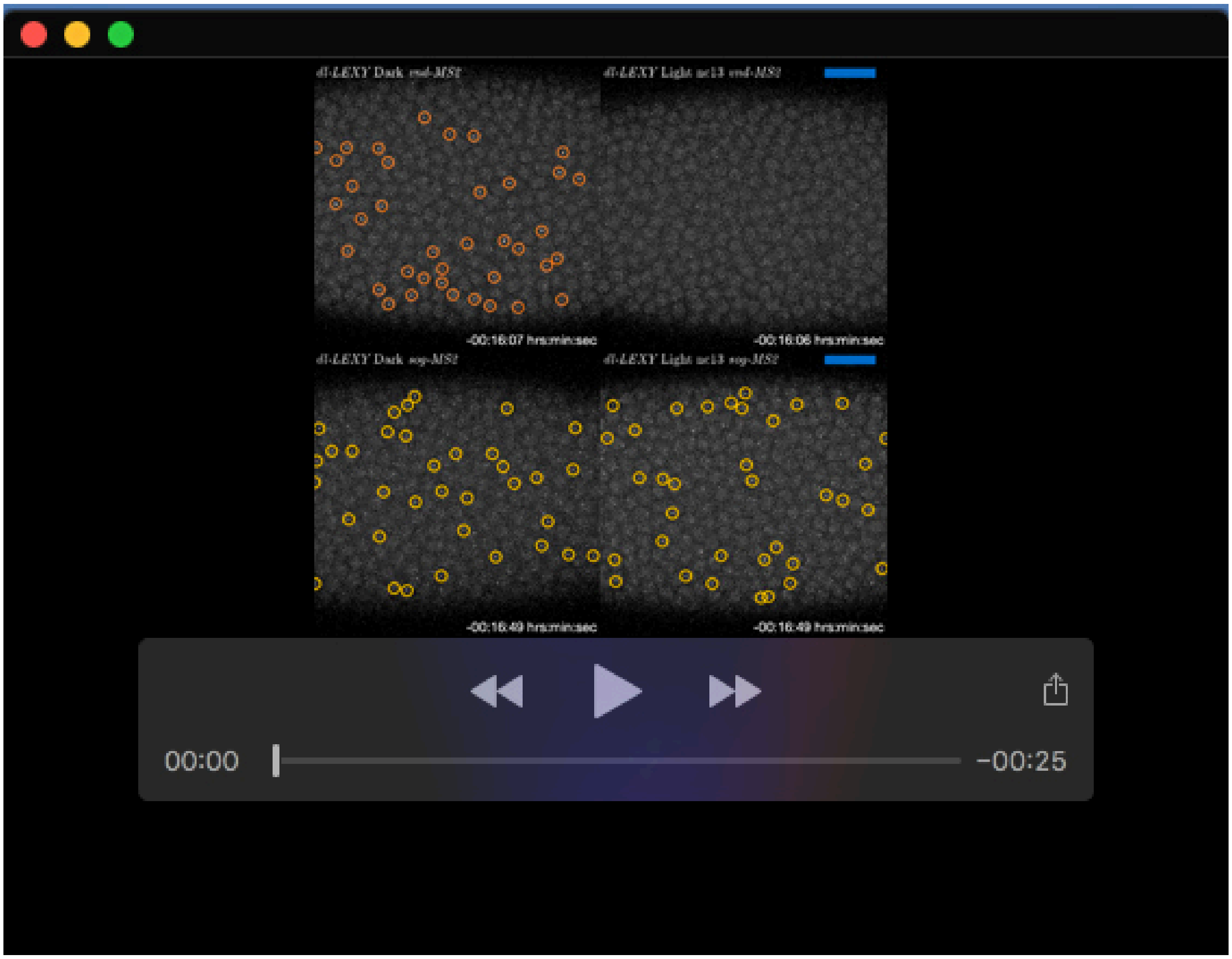

Movie 3. *vndEEE-MS2* (orange) and *sog-MS2* (yellow) in *dl-LEXY* in the dark and with light at nc13. Related to Fig. 2. A ventral view is shown in all panels.

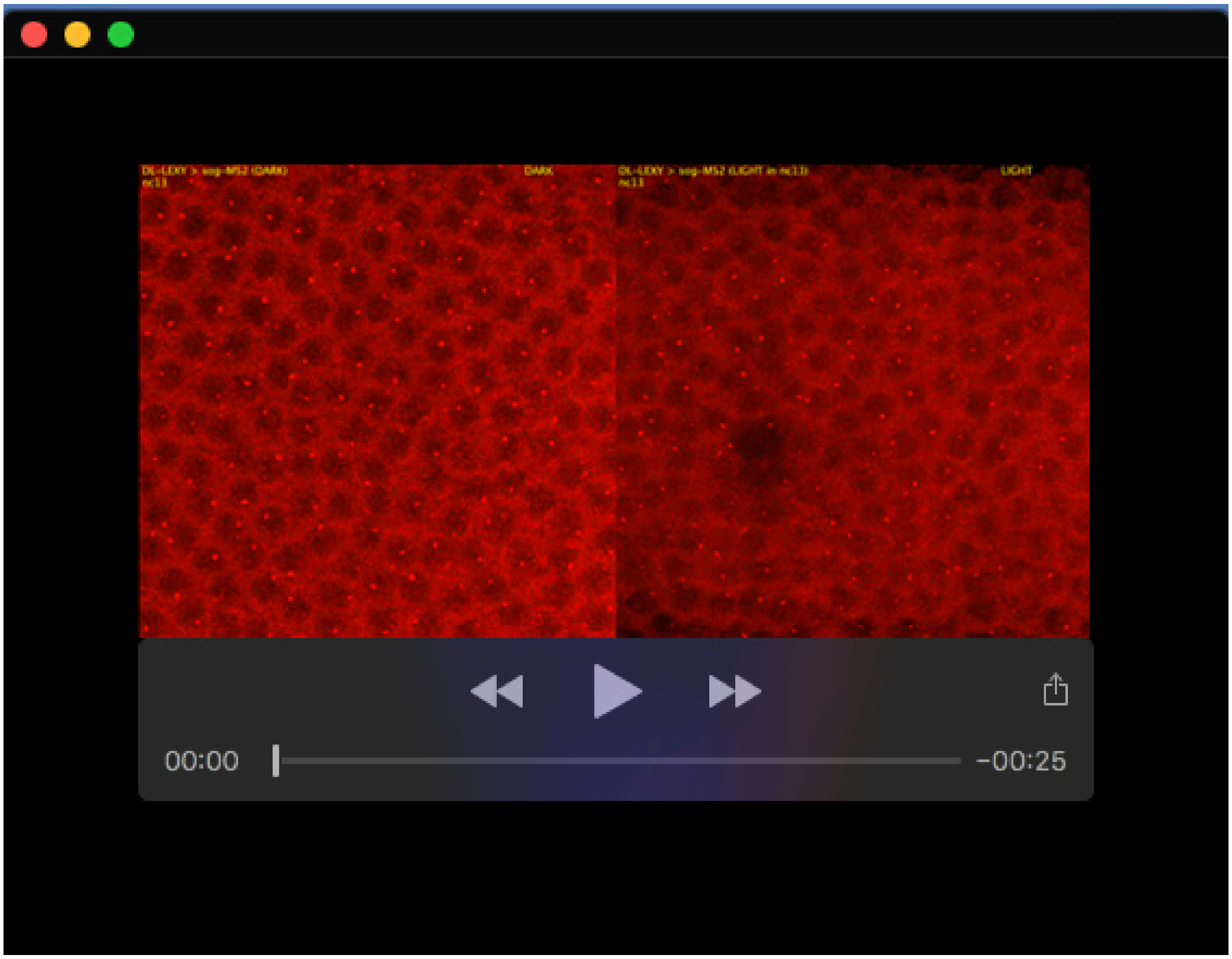

Movie 4. *sog-MS2* in *dl-LEXY* in the dark and with light at nc13, related to Figs 3 and 4.

Views relate to ventrolateral regions of embryos as shown in Fig. 3B.

Development • Supplementary information
